# Application of a centrifugal disc fertilizer spreading system for UAVs in rice fields

**DOI:** 10.1016/j.heliyon.2024.e29837

**Published:** 2024-04-16

**Authors:** Hongyang Zhou, Weixiang Yao, Dongxu Su, Shuang Guo, Ziyue Zheng, Ziqi Yu, Dongyuan Gao, Hongwei Li, Chunling Chen

**Affiliations:** a College of Information and Electrical Engineering, Shenyang Agricultural University, Shenyang 110866, China; b Liaoning Engineering Research Center for Information Technology in Agriculture, Shenyang 110299, China

**Keywords:** Agricultural UAVs, Fertilizer application, Orthogonal test, Centrifugal spreading, Aerospace applications

## Abstract

Unmanned aerial vehicle (UAV) granular fertilizer spreading technology has been gradually applied in agricultural production. However, in the process of spreading operation, the actual influence effect of each factor in field operation is still unclear. Based on the self-developed UAV fertilizer spreading system, this paper explores the effects of three factors, the baffle retraction (B), spreading disc speed (D), and UAV flight altitude (H), on the granular fertilizer spreading effect in the actual field scenarios through the orthogonal test and taking the coefficient of variation (Cv) and relative error of fertilizer application rate (λ) as the evaluation indexes. The results showed that the optimal factor level combination of Cv was 11.23 % for BbDbHa (the baffle retraction is 6 %, spreading disc speed is 600r/min, and UAV flight height is 1.5 m) at UAV flight speed of 2 m/s. The best factor level combination for λ was BbDbHb of 7.99 % (the baffle retraction is 6 %, spreading disc speed is 600r/min, and UAV flight height is 2 m). In addition, by analysing the influence of the weather and the vortex of the rice canopy on the actual spreading effect, it was found that the weather has less influence on the spreading effect of this system, while the vortex caused by the airflow of the UAV rotor has a certain influence on the spreading effect, which is also relatively easy to ignore in fertilizer spreading operations. The results of the study can be used to explore the operational effects of actual fertilizer application by UAVs in rice field, which will help promote the development of UAV spreading technology and provide a reference for precision fertilizer application through agricultural aviation.

## 1 Introduction

In agricultural production, fertilizer granules can provide a continuous supply of various nutrients during the growing process of crops such as rice, which can effectively increase the crop yield [1,2]. However, fertilizers should be used in appropriate amounts; otherwise, crop yields may be reduced [[3], [4], [5]]. Modern precision farming proposes to determine the existing nutrient content of each field and thus direct precise operations that significantly reduce fertilizer use while maintaining yields and reducing environmental impacts [[6], [7], [8]].

In recent years, researchers have worked intensively on fertilizer application technology based on large field patterns and have developed precision farming machinery for easy and fast fertilizer application [9,10]. With the continuous development of fertilizer application technology, large ground-mounted agricultural machinery has significantly increased production efficiency and profitability by carrying various fertilizer application equipment on large fields. Shi et al. [11] evaluated the spreading characteristics of an in-house developed variable speed centrifugal fertilizer spreader by using discrete element simulation and analyzed the relationship between the variation in fertilizer particle distribution and the operating parameters of the spreader. This was followed by tests on an outdoor flat surface, which proved that the developed variable speed spreader had a reduced coefficient of variation and good fertilizer application performance. Alameen et al. [12] modified the fertilizer application rate adjustment system for manual broadcast machines and showed an overall rate error of ±2.6 % through tests; however, some systematic bias occurred when the machine's cylinder was responding, and the fertilizer application accuracy could be further improved. However, there has also been much feedback from studies [13] that have found it difficult to use ground application machinery for certain crops with complex growing conditions, such as rice, which is grown intensively on small plots. Due to the weak root system of rice in the early stages of growth, machines that are used to apply fertilizer in rice fields have poor adaptability, undergo operational difficulties and cause severe crop crushing; thus, applying fertilizer with such machines is difficult and may damage arable land [14].

As an emerging agricultural machine, agricultural drones can adapt to complex terrain and are more suitable for working on small, irregular farmland and hilly areas, reducing soil disturbance and better achieving the purpose of accurate operations [15]. With the continuous development of precision agricultural aerial technology, agricultural drones have been widely used for pest and disease control in major food crops such as wheat [16], rice [17], and maize [18], as well as cash crops such as cotton [19], sugarcane [20], guava [21], betel nut [22], olive [23], pear [24] and citrus [25]. Agricultural UAVs also have a unique range of applications in the field of specialized agricultural operations. In the case of material spreading, for example, agricultural drones reduce damage to crop growth and soil by carrying material spreading devices for various purposes for non-contact operations in the air [26]. Researchers have combined spreading technology with UAV technology to improve the accuracy and effectiveness of spreading devices and control algorithms. Zhang et al. [27] analyzed the effect of different flight speeds and altitudes on the efficiency and quality of aerial spreading of rapeseed and concluded that the flight altitude of UAVs greatly affects the effective spreading width, spreading density and spreading uniformity. Li et al. [28] used an unmanned aircraft with a disc-type seeding device for rice seeding trials and showed through the results of the field trials that the coefficient of variation of the seeding operation was only 12.15 %, which initially proved the feasibility of rice sowing. Yan et al. [29] used UAVs to disperse microparticles to simulate liquid pesticides for pest control and confirmed through two trials that microparticle dispersion could replace liquid pesticides for pest control in the corn field. Zhan et al. [30] used a multi-rotor drone to disperse capsules containing red-eyed wasps, again demonstrating good uniformity of dispersal for effective pest control.

Fertilizer application has also become more feasible with the development of agricultural drone technology [[31], [32], [33]]. At present, centrifugal [34,35], pneumatic [15] and evenly split bars [36] are three of the most commonly used technical methods for spreading small-grain fertilizers. Song et al. [37], proposed a control system for granular fertilizer application by UAV based on prescription maps and evaluated the response speed and fertilizer application accuracy of the system through experiments in rice. A comprehensive analysis method of hierarchical analysis (AHP) is also proposed [38] to compare and analyze the spreading characteristics such as the spreading rate, spreading amplitude and distribution uniformity of four UAV-based granular fertilizer spreaders. The results of the comprehensive analysis show that the pneumatic spreader combined with a slotted wheel spreader has superior performance, and this system has a larger effective strip width and better uniformity. Su et al. [39] developed a centrifugal fertilizer spreading system based on a UAV and obtained a spreading error of 7.30 % through tests, which proved that the spreading system improved fertilizer uniformity and application accuracy. However, actual field validation was missing, since the effects of indoor and outdoor trials were only analyzed in this study. A further search of the literature revealed that most current research on agricultural UAV granular fertilizer application technology is still at the laboratory stage and often lacks a field verification component. The field is the real application scenario for UAV fertilizer application systems, so field trials must be validated.

The orthogonal test is one of the more common and well-established research methods used in field trials. It is generally used for multi-factor and multi-level analyses, which can effectively reduce the number of trials and optimize the ideal factors and level conditions [40]. For example, Zhang et al. [20] conducted an orthogonal test to investigate the distribution of sugarcane spray droplets in the canopy by a quadrotor UAV, obtained combination of the spray volume (15 L/ha), flight height (3 m) and flight speed (4 m/s) as the best factorial conditions and the amount of deposition in each layer of the canopy as the evaluation index. Guo et al. [41] established an orthogonal test table with three factors, the flight parameters (flight speed and altitude), droplet size and crop phenotype, to investigate the effect of droplet dispersions by quadrotor UAVs and determined the amount and uniformity of droplet dispersions as indicators. Based on the above research methods, the orthogonal test approach of this paper also provides useful ideas and references for the evaluation of the practical performance of the UAV centrifugal disc spreading device in the field.

In summary, the aim of this paper is to build on previous research by team member Su and to continue to investigate the effectiveness of the system for field fertilizer application operations [39]. The following three key factors that influence the fertilizer application efficacy are the baffle retraction (B) of the spreading device, the spreading disc speed (D), and the flight height (H) of the UAV. These three variables will help to further clarify the underlying mechanisms of each factor that affects the effectiveness of spreading granular fertilizer. Therefore, the following research objectives are proposed in this work. To optimize the important factors and operational parameters through field trials and to assess the key parameters of the system in terms of fertilizer application accuracy and actual fertilizer application effect using the coefficient of variation and the spreading volume error as evaluation indexes under the through fertilizer application mode of operation.

## 2 Materials and methods

### 2.1 UAV spreading systems

#### 2.1.1 Work platform

**Fig. 1:**
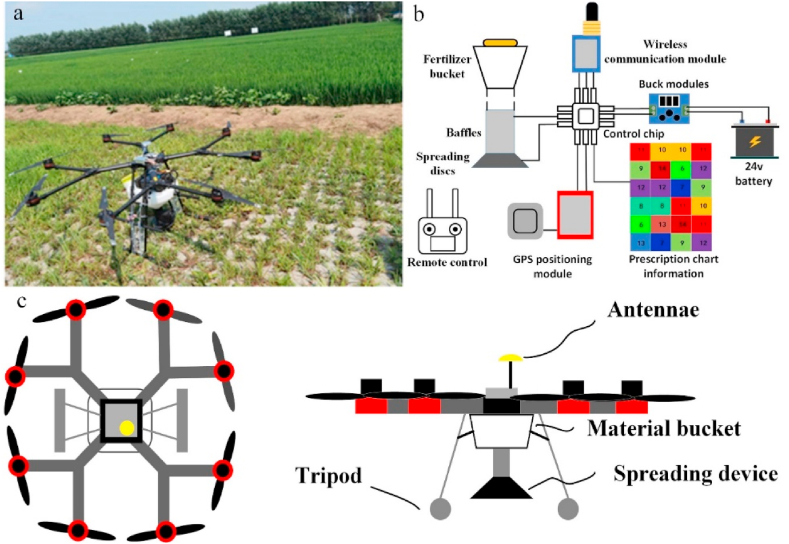
Physical and schematic view of the UAV carrying the spreading system for field trials. (a) Is a physical view of the UAV carrying the spreading system, (b) is a view of the control structure of the spreading system and (c) is a top view of the structure of the UAV with a schematic view of the mounting position of the spreading device.

The MG-1P eight rotor drone (Shenzhen DJI Innovation Technology Co., Ltd.) was modified for fertilizer spraying experiments. A self-developed centrifugal disc fertilizer spraying device was installed below the fuselage (Fig. 1a). Outdoor experiments were conducted by Su et al. [39] at Shenyang Agricultural University Under clear conditions, the temperature ranged from 12 °C to 18 °C, and the average humidity was 5 %. The wind was blowing from the southeast at a speed of less than 2.0 m/s. The evaluation indicators for the outdoor experiments were the coefficient of variation (Cv) and relative error of fertilizer application rate (λ) which were consistent with the results of this field experiment. An orthogonal experimental factor table (Table 1) was used to conduct a 3*3 orthogonal experiment, which preliminarily proved that the system can improve the uniformity and accuracy of fertilization, and has better aerial fertilization performance.

**Table 1:** Orthogonal factor level table for outdoor test.

level	Baffle Opening q/%	Spreading Disc Speed r/min	Flight Height m
1	4	400	1.5
2	6	600	2
3	8	800	3

The spreading system has the STM32F103 as the main control chip, which allows the pre-processed prescription maps to be transferred to the control chip internally via a data storage card. During the operation, the GPS module on the UAV begins to operate, corresponds to the geographical location information in the prescription map and controls the spreading system to carry out the spreading operation at this time by constantly changing the opening and closing degree of the baffle and the spreading disc rotation speed to achieve the purpose of variable spreading according to the instructions. In addition, a manual remote control spreading mode is introduced in this study. Thus, fertilizer can be spread and applied to the field according to the user's operating instructions. Its body position and location will be automatically applied to variable fertilizer application for precise fertilizer application operations.

The control structure of the spreading system is shown in Fig. 1b and consists of a 10 L fertilizer bucket, a master control chip, a wireless communication module, a step-down module, a DC motor and a rudder. The fertilizer bucket is mounted in the centre of the UAV, and the spreading device is suspended from the lower end. This type of connection allows for even forces to be applied to each arm of the drone and maintains the centre of gravity steadily concentrated in the centre, effectively preventing the drone from becoming dangerous during operation. The 24 V battery provides the control chip with a stable operating voltage through the step-down module and powers the whole system. During the system's work, the remote control sends out commands, the wireless communication module on the spreading system receives the signal and begins to operate, and the GPS positioning module starts to locate and identify the latitude and longitude information and carry out variable spreading operations according to the information in the prescription chart. Fig. 1c shows a schematic diagram of the overall structure of the system, with a top view on the left and a front view on the right, allowing a clear view of the position of the spreading device suspension.

#### 2.1.2 Fertilizer spreading model

**Fig. 2:**
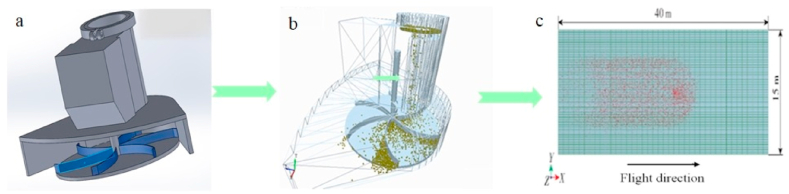
EDEM simulation process diagram.: (a) shows the 3D model of the spreading structure. (b) Shows the movement of the particles in the spreading device, and (c) shows the simulated spreading range of the spreading device.

To obtain more accurate information on the movement of the granular fertilizer during operation, EDEM software (DEM-Solutions, UK) was used to build a spreading and fertilizer application model by the discrete unit method to simulate and analyze the granular spreading movement process and to study the influence of the granular fertilizer distribution and the UAV spreading device to improve the uniformity of the fertilizer application by the spreader. A solid model of the centrifugal fertilizer application unit is constructed using parametric 3D modelling software according to the design dimensions of 1:1, and the corresponding virtual drive and constraint assembly is carried out. The model is simplified according to the state of motion of the operation process, all fixed parts are set into groups, and the dynamics of the moving parts are set individually to speed up the simulation calculation process. The assembled components are shown in Fig. 2a.

In the process of spreading, the horizontal spreading of fertilizer granules causes friction and collision between the granules, thus generating friction forces. In Fig. 2b and Fig. 2c, by simulating the fertilizer application process of the whole fertilizer application system, analyzing the distribution of the group of fertilizer particles, the change of force and the change of displacement, and setting various parameters of the particles, the EDEM simulation shows that the design has a good fertilizer application effect. The simulation shows that the distribution of fertilizer particles has good longitudinal uniformity but poor transverse uniformity.

### 2.2 Field trial

#### 2.2.1 Trial conditions

**Fig. 3:**
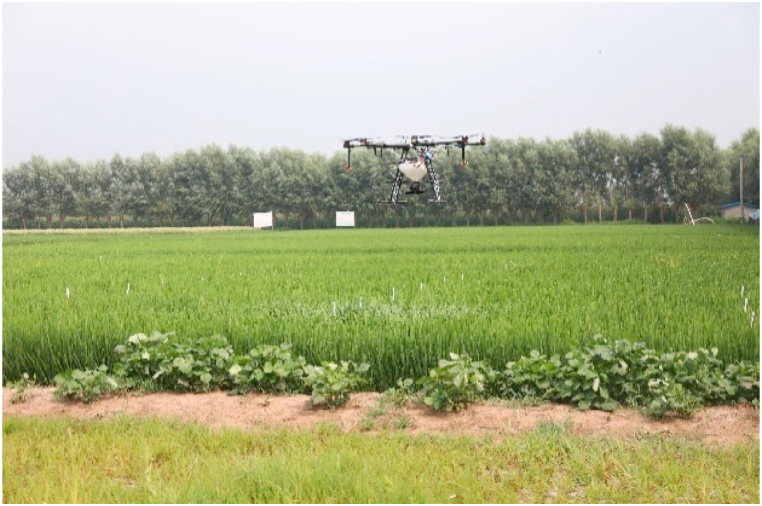
UAV rice fertilizer application operation map.

The field trial was conducted at the Haicheng Experimental Base of Shenyang Agricultural University, Haicheng City, Anshan City, Liaoning Province (40° 98′61.38″ N, 122° 73′68.34″ E). The drone fertilizer application operation scenario is shown in Fig. 3. The trial site is located at mid-latitudes on the east coast of the Asian continent on flat terrain with fertile soil, and the rice variety grown is Panjin V (a variety widely grown in northern China). Prior to the trial, the granular fertilizer left over from previous trials was first removed from the drone as well as from the spreading system and collection bucket. To eliminate the influence of external factors on the experiment, rice fields with average growth were selected for the experiment. The test material chosen for the experiment was a commonly used compound granular fertilizer (Shenyang Zhengmishi Agrochemical Co., Ltd.) with a moisture content of 1.03 %, a mean granule diameter of 4.02 mm and density of 1.36 g/cm^3^.

#### 2.2.2 Orthogonal test design

The orthogonal test determines the significance level of each factor in the test, thus determining the factor priority and the best solution. This field trial was designed through the analysis and conclusions of previous outdoor trials of this research spreading system. To further validate the effectiveness of the spreading system in field trials, the coefficient of variation and the relative error in fertilizer application were selected as evaluation indicators. Considering the operation efficiency and fertilizer application effect, and combining the experience of the outdoor test set the flight speed to 2 m/s. Moreover, three influencing factors, namely, the baffle retraction, spreading disc speed and UAV flight height, were investigated. Nine unidirectional flight treatments were designed for this trial, on which the two best combinations of factors were selected and two further reciprocal test treatments were designed (Table 2). Each test treatment was repeated three times to reduce the generation of test errors and to make the test data more accurate and reliable.

**Table 2:** Experimental design table.

Treatments	Flight Method	Flight speed (m/s)	Retraction of Baffle (%)	Spreading Disc Speed (r/min)	Flight Height (m)
T1	Unidirectional flight mode	2	4	400	1.5
T2	Unidirectional flight mode	2	4	600	2
T3	Unidirectional flight mode	2	4	800	3
T4	Unidirectional flight mode	2	6	400	3
T5	Unidirectional flight mode	2	6	600	1.5
T6	Unidirectional flight mode	2	6	800	2
T7	Unidirectional flight mode	2	8	400	2
T8	Unidirectional flight mode	2	8	600	3
T9	Unidirectional flight mode	2	8	800	1.5
T10	Reciprocating flight mode	2	Bn	Dn	Hn
T11	Reciprocating flight mode	2	Bm	Dm	Hm

Note: Footnotes are n and m are the two best factors selected.

Regarding the nine unidirectional treatments (T1-T9), a 3-factor, 3-level orthogonal test was designed using the baffle retraction, spreading disc speed and UAV flight height as factors. The three levels of baffle expansion are Ba (4 %), Bb (6 %) and Bc (8 %), as shown in Table 3. The three levels of the spreading disc speed are Da (400 r/min), Db (600 r/min) and Dc (800 r/min). The three levels of the drone flight heights are Ha (1.5 m), Hb (2 m) and Hc (3 m). Importantly, the UAV flight height in this study refers to the height of the UAV spreading disc from the crop canopy.

**Table 3:** Test factors and levels.

Level	Factor		
	Retraction of baffle (%)	Spreading disc speed (r/min)	Flight height(m)
1	Ba	Da	Ha
2	Bb	Db	Hb
3	Bc	Dc	Hc

#### 2.2.3 Sampling point arrangement

**Fig. 4:**
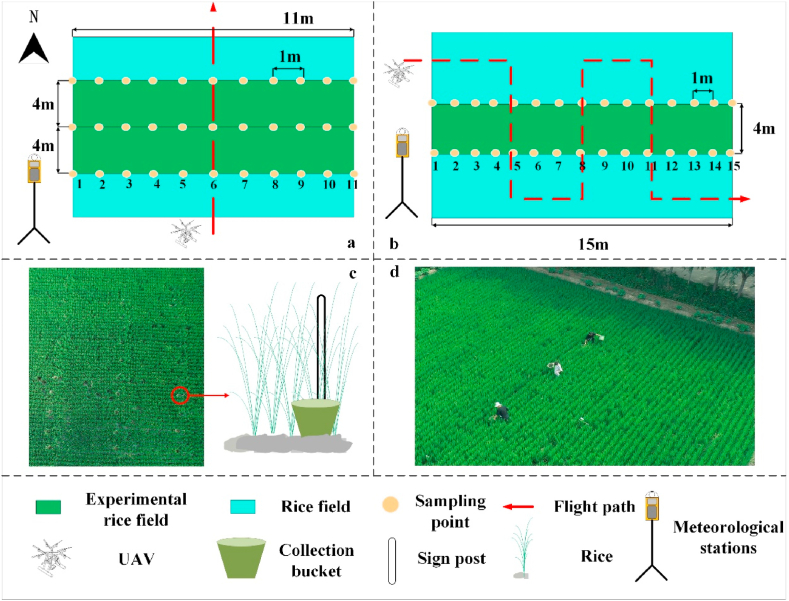
Schematic diagram of the experimental arrangement: (a) shows the experimental arrangement for the unidirectional treatment, (b) shows the experimental arrangement for the reciprocating treatment, (c) shows the position of the collection bucket placement and (d) shows a photograph of the test field arrangement during the field trial of the spreading system.

In the unidirectional test treatments (T1-T9), 33 collection buckets (34 cm diameter) were set up as sampling points in a 3*11 matrix in selected rice test plots. To facilitate the collection and identification of the location of each collection bucket, white marking poles of 1 m in length were placed next to each collection bucket in the arrangement shown in Fig. 4 a. The interval between the two adjacent columns is 4 m, and the spacing between the adjacent sampling points is 1 m. For the purpose of data analysis and processing, the position of the first collection bucket on the left in the schematic is set as Sampling Point 1, and the last point on the right is set as Sampling Point 11. The UAV with the fertilizer application device will spread the fertilizer along the centre line of the sampling point, flying at the altitude and speed set in Table 1, performing the fertilizer application operation and recording the weather data in real time during each trial via a Kestrel 5500 Link micro-weather station (Nielsen-Kellerman, Minneapolis, Minnesota, USA). In the reciprocating test treatment (T10-T11), 30 collection buckets are set up according to a 2*15 matrix, with sign posts placed next to the buckets. Additionally, in the reciprocating test treatment, the position of the first collection bucket on the left in the schematic was set as Sampling Point 1, and the position of the last bucket on the right was set as Sampling Point 15. The two adjacent columns were spaced 4 m apart, and the adjacent sampling points were spaced 1 m apart. The UAV performed the fertilizer application operation at a speed of 2 m/s, following the red flight path that is shown in Fig. 4 b. The collection buckets were retrieved in turn after each test, and the scales were used to separately measure and record the mass of fertilizer collected therein using a balance.

### 2.3 Trial evaluation indicators

According to the specific requirements for the performance of agricultural machinery in GB/T 5262–2008 ″General Provisions for the Determination of Test Conditions for Agricultural Machinery” and the test methods for centrifugal spreaders as specified by ISO 5690, each treatment was repeated three times, and the average of the three trials was taken. The coefficient of variation (Cv) of the particle distribution and the fertilizer application error (λ) were used as the two performance evaluation indicators of the system. The coefficient of variation is used to observe the application error of the spreading system and the uniformity of the fertilizer application, with a smaller coefficient of variation representing a more uniform spreading effect. The fertilizer application error reflects the accuracy of spreading, with a reduced application error representing more accurate spreading of granular material.

The coefficient of variation is calculated by means of the following equation (1):

(1) $$\text{Cv}=\frac{\sqrt{\frac{1}{n-1}\sum {\left(P_{n}-\bar{p}\right)}^{2}}}{\bar{p}}\times 100\%$$

n denotes the number of trials; Pn represents the weight of the collected granular fertilizer for the nth trial; and $\bar{p}$ is the average of the weight of the granular fertilizer per unit of time.

Formula (2) can be used for calculating the relative error λ in fertilizer application:

(2) $$\lambda =\frac{\left|Q-G\right|}{G}\times 100\%$$

λ is the fertilizer application error per unit area, %; Q is the total mass of fertilizer granules per unit area, g/m^2^; and G is the theoretical target fertilizer application rate, g/m^2^.

### 2.4 Data processing

Data were processed using analysis of variance (ANOVA) to assess the coefficient of variation and the fertilizer application error. The overall mean was first calculated, followed by the sum of squares of the between-group, within-group and total coefficients of variation. The degrees of freedom, mean square and F-values were also calculated. SPSS 27.0 (IBM Corporation) and Origin 2021 (OriginLab) were used to conduct joint data analysis, including the creation of orthogonal test tables and conducting an analysis of variance. These statistical methods can be applied to effectively assesses the significance of the test results while helping to ensure rigorous scientific research and improving the reliability and validity of the findings.

## 3 Result

### 3.1 Meteorological and flight parameters

The flight control system on board the agricultural drone recorded the flight parameters during the field trials, while a miniature weather station on the ground recorded the weather data in real time at 2 s intervals during the trials. Table 4 shows the summary statistics of the flight parameters and meteorological data for each treatment test replication. The table shows that the average flight speed for each treatment flight is in the range of 1.8–2.2 m/s. Meanwhile, the average temperature during the test period was 22.91 °C, and the wind speed reached a minimum of 1.8 m/s and a maximum of 2.2 m/s. These results indicated that the wind speed rating was of Class 2 (1.6 m/s∼3.3 m/s), which made it suitable for spreading operations.

**Table 4:** Data sheet on field spreading operations.

Treatment	Average wind speed (m/s)	Average temperature (°C)	Average flight speed (m/s)	Average flight height(m)
T1	2.35 ± 0.14	23.0 ± 0.2	1.9 ± 0.11	1.5 ± 0.2
T2	2.17 ± 0.12	21.5 ± 0.1	2.0 ± 0.09	2.0 ± 0.1
T3	2.52 ± 0.08	24.3 ± 1.1	2.0 ± 0.05	3.0 ± 0.2
T4	2.23 ± 0.04	22.7 ± 0.8	2.1 ± 0.07	3.0 ± 0.2
T5	2.37 ± 0.06	23.5 ± 0.5	2.1 ± 0.09	1.5 ± 0.1
T6	2.42 ± 0.10	24.0 ± 0.9	1.8 ± 0.05	2.0 ± 0.1
T7	2.56 ± 0.12	23.6 ± 0.7	1.9 ± 0.07	2.0 ± 0.2
T8	2.51 ± 0.05	22.5 ± 1.3	2.1 ± 0.10	3.0 ± 0.2
T9	2.38 ± 0.07	22.9 ± 0.9	2.2 ± 0.11	1.5 ± 0.1
T10	2.41 ± 0.11	21.7 ± 0.5	1.8 ± 0.03	1.5 ± 0.1
T11	2.27 ± 0.13	22.3 ± 0.7	2.1 ± 0.06	2.0 ± 0.1

Note: The average flight height is the height of the UAV spreading disc from the crop canopy.

### 3.2 Coefficient of variation analysis

Through Table 5 and the calculation of the extreme difference R, the extreme difference value of B (Retraction of baffle) was determined to be 3.88, and its influence degree was larger than the two factors D (Spreading disc speed) and H (Flight height). Its influence order can be initially determined to be B > D > H. Combined with the results of the orthogonal tests on the coefficient of variation, the smallest value of the coefficient of variation (only 11.23 %) was achieved when the baffle retraction was 6 %, the spreading disc speed was 600 r/min and the UAV flight height was 1.5 m, i.e., at this combination of BbDbHa. Moreover, the best level of fertilizer uniformity was also achieved with these factors. In the analysis of coefficient of variation using SPSS software, the coefficient of determination R^2^ was found to be 0.965, which indicates that the model is well fitted and there is a strong linear relationship between the independent and dependent variables. It was also found in the analysis of variance that the significance of the B term (p = 0.045) was less than 0.05, proving the significant effect of B on the coefficient of variation in this trial.

**Table 5:** Coefficient of variation orthogonal test table.

Treatment	Retraction of baffle	Spreading disc speed	Flight height	Cv
T1	Ba	Da	Ha	15.71
T2	Ba	Db	Hb	13.73
T3	Ba	Dc	Hc	14.82
T4	Bb	Da	Hc	13.47
T5	Bb	Db	Ha	11.23
T6	Bb	Dc	Hb	12.81
T7	Bc	Da	Hb	17.67
T8	Bc	Db	Hc	16.35
T9	Bc	Dc	Ha	15.14
F	21.323	5.138	1.171	
Significance	0.45^a^	0.163	0.461	
Square	22.811	5.497	1.253	
Degree of freedom	2	2	2	
Mean Square	11.405	2.748	0.626	
Order	Retraction of Baffle > Spreading Disc Speed > Flight Height			
Optimal combination	BbDbHa			

Cv	k_1_	14.75	15.62	14.03
	k_2_	12.50	13.77	14.74
	k_3_	16.39	14.26	14.88
	R	3.88	1.85	0.85

Note.

a stands for significant; Ba, baffle retraction of 4 %; Bb baffle retraction of 6 %; Bc baffle retraction of 8 %; Da, spreading disc speed of 400r/min; Db, spreading disc speed of 600r/min; Dc spreading disc speed of 800r/min; Ha, UAV flying height of 1.5 m; Hb, UAV flying height of 2 m; Hc, UAV flying height of 3 m

The three factors all had some influence on the coefficient of variation, with the greatest influence being the amount of baffle retraction (F = 21.323), followed by the spreading disc speed (F = 5.138). The least influential factor was the UAV flight height (F = 1.171). The two treatments used as Bb were T4 and T6, both with coefficients of variation of 13.47 % and 12.81 %, respectively, which were larger than the values of the coefficient of variation for the T5 treatment. There was an increase in the coefficient of variation for each Ba (T1, T2 and T3) treatment, whose mean coefficient of variation was 14.75 %, which is 2.25 % more than that of Bb (T4, T5 and T6). Moreover, there was a significant increase in the coefficient of variation for each Bc (T7, T8 and T9) treatment, whose mean coefficient of variation was 16.38 %, which is 3.88 % more than that of Bb (T4, T5 and T6). Thus, the effect of the baffle retraction at different levels had a greater effect on the coefficient of variation.

The mean coefficients of variation for the Da (T1, T4 and T7), Db (T2, T5 and T8) and Dc (T3, T6 and T9) treatments were 15.62 %, 13.77 % and 14.26 %, respectively. It can be seen that Db has the lowest coefficient of variation, followed by Dc and Da, which had the highest average coefficient of variation. Therefore, a spreading disc speed of 600 r/min was better than the other two speeds, which also indicates that too high or too low a spreading disc speed caused an increase in the coefficient of variation.

The mean coefficients of variation for the Ha (T1, T5 and T9), Hb (T2, T6 and T7) and Hc (T3, T4 and T8) treatments were 14.02 %, 14.73 % and 14.88 %, respectively, which clearly showed that the mean coefficient of variation increased with an increasing UAV altitude. Therefore, the higher the UAV altitude is, the more particulate matter that was affected in the air. This suggests that the higher the altitude of the drone is, the greater the impact on the particulate matter in the air, which in turn suggests that the altitude of the drone should not be too high for spreading operations.

**Fig. 5:**
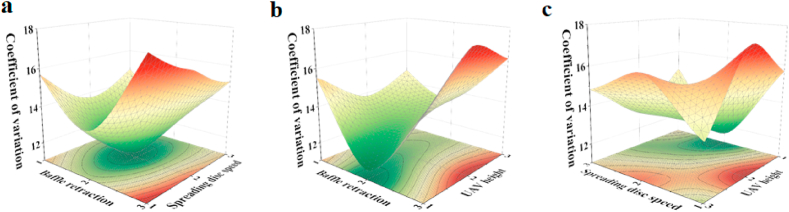
Response surface plot of the coefficient of variation: (a) the response surface plot of the baffle retraction vs. the spreading disc speed vs. the coefficient of variation, (b) the response surface plot of the baffle retraction vs. the UAV flight height vs. the coefficient of variation, and (c) the response surface plot of the spreading disc speed vs. the UAV flight height vs. the coefficient of variation.

To clarify the changes in the coefficient of variation by each influencing factor, the response surface of the coefficient of variation was plotted (Fig. 5). The surface variation in the response of the baffle retraction and spreading disc speed to the coefficient of variation can be seen more clearly in Fig. 5a. Specifically, the coefficient of variation decreased by an average of 2.21 % and then increased by an average of 1.34 % as the spreading disc speed increased at baffle retractions of 4 % and 6 %. At a baffle retraction of 8 %, the coefficient of variation decreased by 1.32 % and then decreased by 1.12 % as the spreading disc speed increased from 400r/min to 800r/min. However, for the same spreading disc speed, the coefficient of variation consistently shows a decreasing and then increasing trend, with the baffle retraction having a faster rate of change on the coefficient of variation. This is probably because the lower the spreading disc speed is, the more uneven the particle distribution and the higher the coefficient of variation of the distribution. Additionally, the density of the contour lines, which represents a greater effect of the spreading disc speed on the coefficient of variation than the amount of baffle retraction on the coefficient of variation, was analyzed.

Fig. 5b shows a response surface plot of the baffle retraction and UAV flight altitude. The coefficient of variation decreased from 15.71 % to 13.73 % at a baffle retraction of 4 % and then gradually increased to 14.82 %. At a baffle retraction of 6 %, the coefficient of variation increased with an increasing UAV flight altitude. At 8 %, the coefficient of variation increased by 2.53 % and then decreased by 1.32 %. This demonstrates that for a given amount of baffle retraction, the UAV flight altitude had a slower change on the coefficient of variation, indicating that the amount of baffle retraction had a more significant effect on the coefficient of variation. The coefficient of variation showed an average decrease of 2.25 % and then an increase of 3.89 % as the baffle retraction increased for UAV flight heights of 1.5 m, 2 m and 3 m, respectively. Based on the contour distribution, the denser contours represent a deeper influence, indicating that the UAV flight height had a weaker effect on the rate of change of the coefficient of variation relative to the amount of baffle retraction.

Fig. 5c shows a response surface plot of the variation in the spreading disc speed versus the UAV flight altitude. The coefficient of variation decreased with the decrease in the spreading disc speed when the UAV flight height was 1.5 m and then increased. Moreover, the coefficient of variation decreased with the increase in the spreading disc speed when the UAV flight height was 2 m. Finally, when the UAV flight height was 3 m, the decrease in the spreading disc speed showed that the change in the coefficient of variation increased and then decreased. The coefficient of variation of the spreading disc speed varies at a certain altitude. When the spreading disc speed was 400 r/min, the coefficient of variation increased from 15.71 % to 17.67 % and then decreased to 13.47 % as the UAV flight altitude increased. When the spreading disc speed was 600 r/min, the coefficient of variation continued to increase with the UAV flight altitude. However, at a spreading disc speed of 800 r/min, the coefficient of variation decreased from 15.14 % to 12.81 % and continued to rise to 14.82 %. This was probably because the lower the spreading disc speed was, the lower the centrifugal force acting on the particles, the lower the effective bar width and the lower the UAV flight altitude, which likewise decreased the UAV spreading uniformity. Analysis of the contours showed that for a given spreading disc speed, the UAV flight altitude had a greater influence on the coefficient of variation than the spreading disc speed.

From the analysis of the above table and the response surface plots, it can be concluded that under the evaluation index of the coefficient of variation, the amount of baffle retraction was significant for the variation in the coefficient of variation, followed by the spreading disc speed and finally the UAV flight height. These results are consistent with the pattern summarized in the ANOVA table. Compared to the optimal parameters derived from the outdoor trials, the coefficient of variation of the optimal parameters from the field trials increased by 1.43 %, and the spreading uniformity of the spreading system decreased.

### 3.3 Analysis of the relative error in the fertilizer application rates

From Table 6 and the calculation of the extreme difference (R), a value of 2.18 was calculated for the extreme difference in the spreading disc speed, which was greater than the other two factors (B and H). This showed that the influence of the spreading disc speed is greater than that of the two factors B and H. The order of influence could be tentatively determined to be D > B > H. Based on the test results, the minimum fertilizer application error (only 7.33 %) was achieved when using a combination of BaDbHb conditions, i.e., a 4 % baffle retraction, a spreading disc speed of 600 r/min and a UAV flight height of 2 m. The coefficient of determination (R^2^) of the fertilizer application error was 0.911, which was obtained after analysis with the SPSS software. This result indicates that a minor experimental error occurred. After ANOVA, it was found that the significance of all three factors was greater than 0.05, which could be due to the influence of environmental factors.

**Table 6:** Fertilizer application error orthogonal test table.

Treatment	Retraction of baffle	Spreading disc Speed	Flight height	λ
T1	Ba	Da	Ha	11.31
T2	Ba	Db	Hb	7.33
T3	Ba	Dc	Hc	10.81
T4	Bb	Da	Hc	9.34
T5	Bb	Db	Ha	7.99
T6	Bb	Dc	Hb	9.72
T7	Bc	Da	Hb	10.87
T8	Bc	Db	Hc	10.18
T9	Bc	Dc	Ha	11.52
F	3.359	5.805	1.057	
Square	5.107	8.825	1.606	
Degree of Freedom	2	2	2	
Mean Square	2.554	4.412	0.803	
Order	Spreading Disc Speed > Retraction of Baffle > Flight Height			
Optimal combination	BbDbHb			

λ	k_1_	9.82	10.51	10.27
	k_2_	9.02	8.50	9.31
	k_3_	10.86	10.68	10.11
	R	1.84	2.18	0.97

Note: Ba, baffle retraction of 4 %; Bb baffle retraction of 6 %; Bc baffle retraction of 8 %; Da, spreading disc speed of 400r/min; Db, spreading disc speed of 600r/min; Dc spreading disc speed of 800r/min; Ha, UAV flying height of 1.5 m; Hb, UAV flying height of 2 m; Hc, UAV flying height of 3 m.

Table 6 shows that the spreading disc speed has the greatest effect on the fertilizer application error (F = 5.805) and that the other two baffle retractions (F = 3.359) and the UAV flight altitude (F = 1.057) both have a limited effect on the fertilizer application error. In addition, for the two treatments T5 and T8 of Db, the fertilizer application error was 7.99 % and 10.18 %, with an increase in the fertilizer application error of 0.66 % and 0.82 %, respectively, compared to the values of the T3 treatment. A further summary of the spreading disc speed treatments showed that the fertilizer application error was higher for each Da (T1, T4 and T7) and Dc (T3, T6 and T9) treatment, with an average fertilizer application error of 10.51 % and 10.68 %, respectively. These errors were greater than the average fertilizer application error at Db.

The average fertilizer application errors for the Ba (T1, T2 and T3), Tb (T4, T5 and T6) and Tc (T7, T8 and T9) treatments were 9.82 %, 9.01 % and 10.86 %, respectively. Bb had the lowest mean fertilizer application error, followed by Ba and Bc, which had the highest mean fertilizer application error. The average fertilizer application error for each Ha (T1, T5 and T9), Hb (T2, T6 and T7) and Hc (T3, T4 and T8) treatment was 10.27 %, 9.31 % and 10.11 %, respectively. Again, Hb had the smallest average fertilizer application error, and Ha and Hc had the largest average fertilizer application error.

**Fig. 6:**
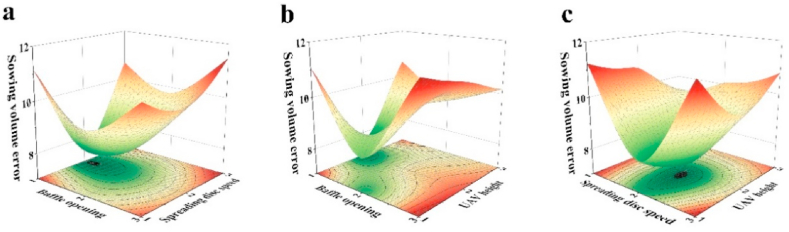
Response surface plot of the relative error during fertilizer application: (a) the response surface plot of the baffle retraction versus the spreading disc speed on the relative error during fertilizer application, (b) the response surface plot of the baffle retraction versus the UAV flight height on the relative error during fertilizer application, and (c) the response surface plot of the spreading disc speed versus the UAV flight height on the relative error during fertilizer application.

To more clearly clarify the variation in each influencing factor on the fertilizer application error, a response surface plot of the coefficient of variation was drawn (Fig. 6). Fig. 6a shows the surface variation of the response of the baffle retraction and spreading disc speed on the fertilizer application error. When the baffle retraction was constant, the fertilizer application errors all showed an average decrease of 2.01 % and then an increase of 2.18 % as the spreading disc speed increased. At spreading disc speeds of 400 r/min and 800 r/min, the fertilizer application error also showed a decrease of 0.53 % and then an increase of 1.66 % as the baffle retraction increased; however, at 600 r/min, the application error decreased as the baffle retraction decreased. This might be due to a decrease in the spreading speed of the spreading disc, which leads to a decrease in the spreading accuracy of the granular fertilizer. This indicates that at a certain spreading disc speed, the baffle retraction had a smaller effect on the rate of change of the fertilizer application error with a slower contour distribution, thus proving that the baffle retraction has a weaker effect on the fertilizer application error.

Fig. 6b shows a surface plot of the response of the baffle retraction and UAV flight altitude on the fertilizer application error. When the UAV was flown at 1.5 m and 3 m, the fertilizer application error decreased by 2.39 % and then increased by 2.18 % as the baffle retracted, and at 2 m, the fertilizer application error increased as the baffle retracted. The analysis of the contour lines also showed that for the same UAV flight altitude, the fertilizer application error at different baffle retractions mostly shows a decreasing and then increasing trend, indicating that the baffle retractions had a deeper impact on the fertilizer application error compared to the UAV flight altitude. At a baffle retraction of 4 %, the fertilizer application error decreased from 11.31 % to 7.33 % and finally to 10.81 % with an increasing UAV altitude; however, at a baffle retraction of 6 %, the fertilizer application error increased from 7.99 % to 9.72 % and finally to 9.34 % with an increasing UAV altitude. At a baffle retraction of 6 %, the fertilizer application error increased with the UAV altitude from 7.99 % to 9.72 % and finally decreased to 9.34 %. At a baffle retraction of 8 %, the fertilizer application error increases with a decreasing UAV altitude. This was probably because the lower the baffle retraction was, the lower the amount of granular fertilizer deposited at the outlet, and the UAV flight height affected the accuracy of spreading. This results in a slower contour distribution of fertilizer for the same level of baffle expansion and a different trend of fertilizer application error at different UAV flight heights, demonstrating the weak influence of the UAV flight height on the fertilizer application error.

Fig. 6c shows the response surface variation of the spreading disc speed and UAV flight altitude with respect to the fertilizer application error. When the spreading disc speed was 600 r/min and 800 r/min, the fertilizer application error decreased by 1.23 % and then increased by 1.97 % with an increasing UAV flight height. At a spreading disc speed of 400 r/min, the spreading disc speed decreased with an increasing UAV flight height. For the same spreading disc speed, the fertilizer application errors for different UAV flight altitudes mostly showed a decreasing trend followed by an increasing trend, while a few showed an increasing trend all the time. When the UAV flight height was 1.5 m and 2 m, the fertilizer application error decreased by 3.43 % and then increased by 2.92 % as the spreading disc speed decreased. When the UAV flight height was 3 m, the fertilizer application error decreased as the spreading disc speed decreased. This might be because a high UAV flight altitude may be influenced by environmental factors, resulting in a decrease in the spreading accuracy. Moreover, this result suggests that the variation in the spreading disc speed relative to the UAV flight altitude had a greater effect on the amount of fertilizer applied error.

The analysis of the variance of the fertilizer application error and the corresponding three response surface plots led to the conclusion that the influence of the spreading disc speed on the fertilizer application error was more pronounced than the influence of the baffle retraction and the flight height on the relative fertilizer application error, followed by the baffle retraction and finally the flight height of the UAV. These results are consistent with the pattern summarized in the orthogonal test table. Compared to the optimal parameters of the outdoor trials and the optimal parameters of the field trials (BbDbHb), the adjusted fertilizer application error increased by 4.23 %.

### 3.4 Comparison of the field and outdoor trial data

**Fig. 7:**
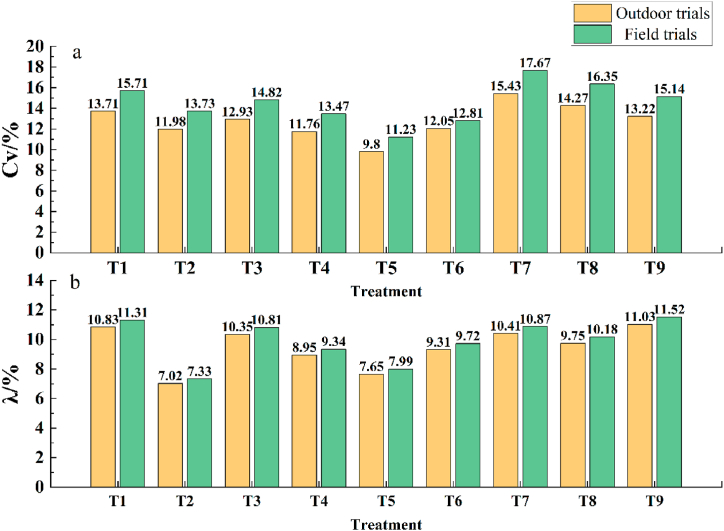
Coefficient of variation and the relative error of fertilizer application rate for outdoor (Su et al. [39]) and field trials: (a) Coefficient of variation in outdoor and field trials, (b) the relative error of fertilizer application rate in outdoor and field trials.

Fig. 7 Compares the data and conclusions from the previous outdoor trial with those from the current field trial. The coefficients of variation and errors of relative errors of fertilizer application are shown in Fig. 7 a. and Fig. 7 b. for the two experiments, with each treatment corresponding to the same influencing factors and level parameters. The results indicate that the overall values of the outdoor trial were smaller than those of the field trial. The coefficient of variation for the nine treatments in the outdoor trial was 12.79 %, with a relative error of fertilizer application of 9.48 %. Similarly, the coefficient of variation for the nine treatments in the field trial was 14.55 %, with a fertilizer application error of 9.89 %. These findings suggest that the coefficient of variation in the field trial was significantly higher than that in the outdoor trial, with a difference of 1.76 %. However, the variation relative error of fertilizer application rate was relatively low, with a difference of only 0.41 %. Thus, the fertilizer application error and system accuracy were both reduced in the field. The coefficient of variation and relative error of fertilizer application rate were observed separately for both trials, and both showed similar trends. The results indicate that the field fertilizer application operating system developed in this study meets the requirements of practical field operations.

### 3.5 Sampling point dispersal results

**Fig. 8:**
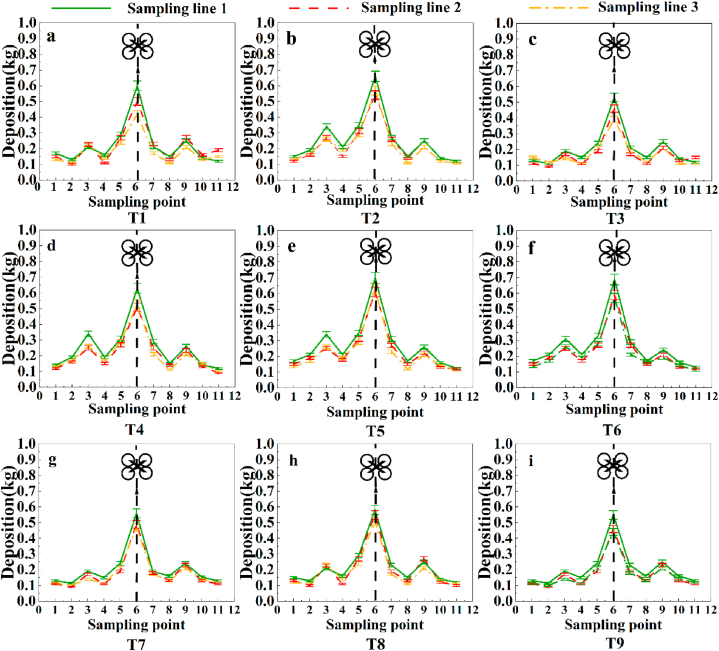
Plot of the granular fertilizer dispersion at each sampling point for the one-way treatment. The collection bucket is a circular bucket with an opening size of 34 cm in diameter.

Fig. 8 shows the distribution of the fertilizer mass in the collection buckets at the sampling points in each collection strip for the nine unidirectional flight treatments, with each line graph showing three replicate trials at the same horizontal level and the values obtained from each trial plotted in the same line graph. From the graph of the granular fertilizer deposition for the unidirectional flight mode in Fig. 8(a–i), it is clear that the variation in the fertilizer collection content of the collection buckets for the three collection strips for the same treatment is indeed relatively close, with the larger dispersion values for each collection strip mostly occurring at the location of the sampling point below the UAV route (6), and the dispersion amounts are more consistent across the sampling points for the three collection strips for the same treatment, with more distribution in the middle. The mean fertilizer mass collected at Sampling Points (4, 8) for Unidirectional Flight Mode T1-T9 was 76.78 g and 93.32 g less than that collected at Sampling Points (3, 9).

**Fig. 9:**
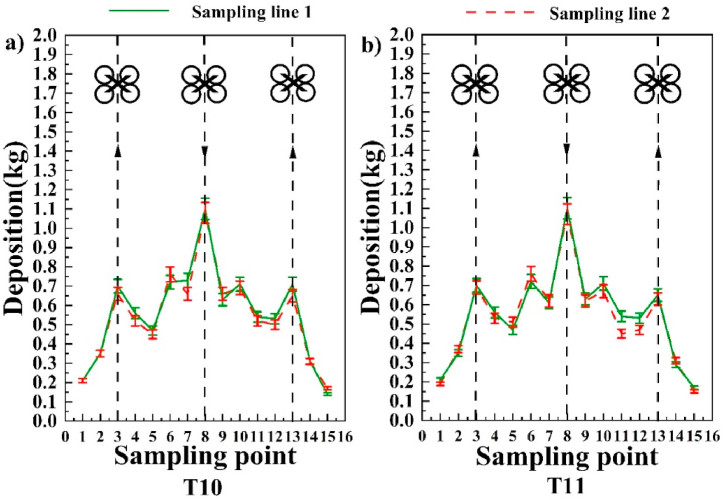
Reciprocating treatment of the granular fertilizer dispersion at each sampling point plotted with a collection drum with an opening size of 34 cm.

The distribution of granular fertilizer dispersion at each sampling point for the reciprocal treatment is shown in Fig. 9. For T10 and T11, the two treatments correspond to Fig. 9a and b respectively, and the sampling point locations (3, 6, 8, 10 and 13) show a greater dispersion under the reciprocal treatments, suggesting that the aircraft tends to have a greater intermediate dispersion in the unidirectional flight mode condition. The sampling points for the reciprocal flight modes (T10, T11) also show a general trend of greater dispersion in the middle and lesser dispersion on the sides. Based on the results of the treatment plots for the two reciprocal flights, it is clear that the large distances between the routes during the UAV operations in the field can lead to significant differences in the dispersion volumes. For example, at Sampling Points (5, 11, 12), the average dispersion amounts of 0.475 kg, 0.513 kg and 0.508 kg were too different from the dispersion amounts at the adjacent sampling points (4, 6, 10, 13) (0.54 kg, 0.74 kg, 0.695 kg and 0.66 kg, respectively), and this difference may have affected the final spreading results. In practice, therefore, the distance between the UAV routes should be properly controlled to make full use of the advantages of UAV spreading and to ensure consistency and stability of the spreading results. However, the overall trend is largely the same as in the trials with direct flight treatments. Combining the results of the unidirectional and reciprocal flight mode revealed that the amount of dispersion at the sampling points in the unidirectional flight mode (4, 5, 7, 8) and the reciprocal flight mode (4, 5, 6, 7, 9, 10, 11, 12) was less than that at the sampling points directly below the route. This phenomenon may be related to the influence of meteorological conditions and eddies during the spreading operations carried out by the UAV. The source of the lower dispersion at the sampling sites will be analyzed in depth in the discussion section of this paper.

## 4 Discussion

In general, the coefficient of variation is less than 15 %, and the spreader can be considered capable of spreading evenly in the sessions of the field trial [38]. However, this system calculates the value of the coefficient of variation in the field trials in which four of the flight treatments had results greater than 15 %, the largest of which was 2.67 % greater than the standard value. In addition, the relative error in fertilizer application was generally less than 10 % in the statistical analysis, which proves that the device performs accurate spreading in the trials [42]. Five of the flight treatments in field trials had results greater than 10 %, the largest of which was 1.52 % higher than the standard. When comparing the outdoor trials with the field trials, in the outdoor environment, one and four values of the coefficient of variation and spreading error each exceeded the evaluation criteria due to natural conditions such as ambient wind. Regarding the actual field trials, the overall coefficient of variation and the relative error in the fertilizer application were higher than the results obtained in the outdoor trials, with the coefficient of variation increasing by 1.76 % and the relative error of fertilizer application rate increasing by 0.41 %. Compared to the weather of the outdoor test and the field test, the weather conditions during the outdoor test were sunny, with an average temperature of 18 °C and wind speed ranging from 1.5 m/s to 1.7 m/s. However, the weather conditions during the field test were cloudy, with an average temperature of 23 °C and wind speed ranging from 2.2 m/s to 2.5 m/s. The comparison of the weather conditions showed that the difference in the wind speed values between the two conditions was significant, indicating that a certain wind speed during the spreading by unmanned aerial vehicles can cause the particles to move with the wind, thus causing certain errors. As determined by the meteorological station, the wind speed of each treatment in this study did not exceed 2.2 m/s, which is within the range that allows UAV operation. Meanwhile, it is generally believed that the wind field of the UAV rotor has a greater influence on the actual spreading effect than the ambient wind, which was confirmed by the vortex in the rice canopy created by the rotor air flow. However, by analyzing the data and comparing the coefficient of variation and the relative error in the fertilizer application between the orthogonal test tables from the outdoor and field trials, it was found that most of the data collected had the same trend even at two different wind speeds, indicating that the system can still meet the requirements of field operations and apply fertilizer at lower wind speed levels without affecting the normal operation of the UAV.

**Fig. 10:**
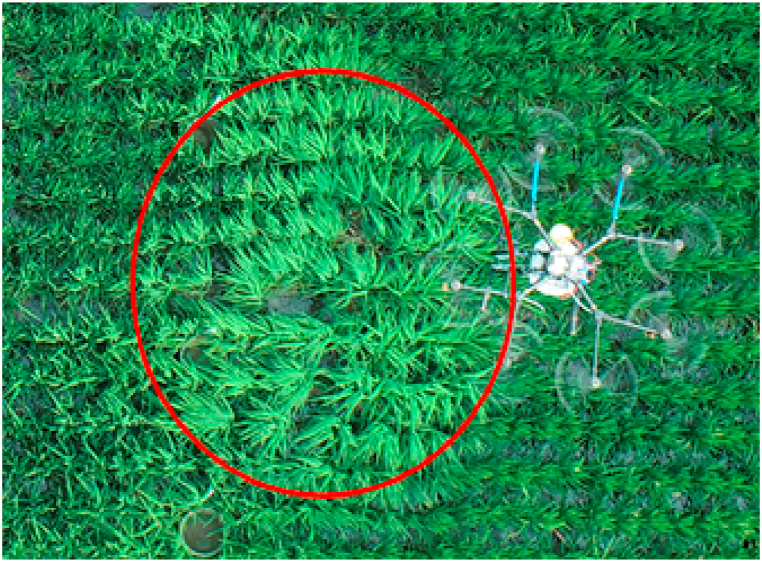
Vortices generated during the field trial.

The most noticeable difference between the outdoor and field trials was the effect on the rice plant, in which the leaves of the rice and the vortex formed by the drone flying over the rice had some effect on the spreading effect. During operation, the UAV pushes down air to suspend itself. To achieve hovering or movement in the air, this downwards force will cause the state of the rice canopy to change, resulting in the crop canopy having a strong rotor airflow blown away, forming a vortex phenomenon in the crop canopy. This process may be due to the shading of the crop canopy causing the collection bucket to fall. This factor modifies the effectiveness of the experiment. Fig. 10 Shows the vortex created by the UAV operation during this experiment. Clearly, the formation of the vortices causes the rice canopy to tilt. When granular fertilizer is thrown from the spreading system, it may hit the rice leaves and cause the granular fertilizer to disperse, reducing the mass of granular fertilizer in the collection bucket and affecting the spreading effect of the spreading system.

However, during the indoor test, which was conducted in the same way as the field test without the influence of the crop canopy, the best parameters were 12.73 % and 4.23 % less in the variation and error in the spreading volume, respectively, than in the field trial. This indicates that the vortex formed during the operation has a greater impact on the UAV spreading operation. Fan et al. used video aerial photography technology to obtain the impact of airflow on rice fields through the take-off mass, flight height and flight speed and established an orthogonal test table to explore the degree of influence of these three factors on the vortex tail distance, the stability of the trailing tail, the area of the vortex tail and the shape of the ranking [43]. The results showed that the UAV flight speed is the most influential factor on the vortex. Thus, the farther the tail vortex distance and the longer the duration, the easier it is for the rice to be disturbed and dissipated, and the less stable it is. Liu et al. developed a honeycomb parameter control model for a quadrotor UAV. There was a significant functional relationship between the flight parameters and the wind vortex parameters [44]. Guo et al. studied the effects of the vortex morphology, droplet particle size and crop phenotype on the drone droplet dispersion and found that the vortex morphology affects the droplet dispersion as well as the uniformity [45]. In summary, vortex formation is related to the UAV's flying speed, altitude and take-off mass, which in turn affects the UAV's stability. In particular, flying the UAV too fast can result in greater airflow disturbance and vortex instability, making the UAV more susceptible to vortex effects. At lower altitudes, where the air is denser, the effects of vortices can be more pronounced. In addition, heavier UAVs generate greater inertia and momentum during launch and flight, which can lead to stronger vortex flows. The combination suggests that the vortices formed by the UAV during operation can have a significant impact on the UAV spreading operation, resulting in an overall large coefficient of variation for the evaluation indicators and relative error values for the fertilizer application in the field trials. The effect of the vortex on the spreading effectiveness should be considered during field trials or field operations, and the vortex morphology should also be considered a factor that influences the effectiveness of the UAV spreading operations in future research.

## 5 Conclusion

This study was carried out by combining the findings of previous studies with EDEM simulations based on the designed spreading system and field trials to verify the spreading effect of fertilizer. Based on the UAV-mounted spreading system, field trials were conducted with the baffle retraction, spreading disc speed and UAV flight height considered to investigate the real-life scenarios of the design in actual field trials and to further confirm the conclusions reached by the system in outdoor trials. The coefficient of variation and the relative error in fertilizer application were analyzed to determine the best solution to achieve fertilizer deposition:

(1) By analysing the results of the orthogonal tests, the three core factors of the baffle retraction (B), spreading disc speed (D) and UAV flight height (H) were found to have the most important effects on the coefficient of variation. The order of importance were as follows: the baffle retraction, spreading disc speed and UAV flight height. The best combination of the factor levels was BbDbHa. Regarding the relative error in the fertilizer application, the spreading disc speed was the most effective, followed by the baffle retraction and UAV flight height, with the best combination of factor levels being BbDbHb. The baffle expansion and spreading disc speed proved to be the most important factors that influence the coefficient of variation and the relative error during fertilizer application. However, there is some variation in the importance of the effect of the UAV flight altitude on the coefficient of variation and the relative error in fertilizer application.
(2) Under the optimum combination of the fertilizer application parameters, the coefficient of variation (Cv) value was 11.23 %, and the relative error in the fertilizer application (λ) value was 7.33 %. Compared to the outdoor trials in the previous study, the overall coefficient of variation in the field trials was greater at 1.76 %, while the relative error in the fertilizer application was less variable at 0.41 %. The test results show an increasing trend in the particle dispersion with an increasing spreading disc speed. During spreading, the effects of meteorological conditions and eddies should be minimized, and the coefficient of variation and relative error on the fertilizer application rates should be accounted for to determine the most suitable solution.

Scientific and rational fertilizer application operations can reduce fertilizer inputs while reducing operating costs and environmental pollution. Moreover, the fertilizer utilization and fertilizer efficiency can be improved, which in turn improves the crop yields, reduces fertilizer usage and costs and improves yields. Compared with other fertilizer application methods, this fertilizer application process has the advantages of being environmentally friendly, efficient and of high quality. Furthermore, this method can provide economical, ecological and social benefits and provides some references for further strengthening the standardized application of aerial fertilizer application technology in precision agriculture.

## Data availability

No data was used for the research described in the article.

## Fund

This research was supported by the Doctoral Scientific Research Foundation of Liaoning Province (2023-BS-123), the General Program of Liaoning Provincial Educational Department (LJKMZ20221059) and the Liaoning Applied Basic Research Program Project (2023JH2/101300120).

## CRediT authorship contribution statement

**Hongyang Zhou:** Writing – original draft, Validation, Methodology, Formal analysis, Conceptualization. **Weixiang Yao:** Writing – review & editing, Supervision, Project administration, Investigation, Funding acquisition. **Dongxu Su:** Writing – review & editing, Investigation. **Shuang Guo:** Visualization, Investigation. **Ziyue Zheng:** Methodology, Data curation. **Ziqi Yu:** Investigation, Data curation. **Dongyuan Gao:** Writing – review & editing. **Hongwei Li:** Supervision, Resources. **Chunling Chen:** Writing – review & editing, Supervision, Funding acquisition.

## Declaration of competing interest

The authors declare that they have no known competing financial interests or personal relationships that could have appeared to influence the work reported in this paper.

## References

[1] Ding S.P., Bai L., Yao Y.X., Yue B., Fu Z.L., Zheng Z.Q., Huang Y.X. (2018). Discrete element modelling (DEM) of fertilizer dual-banding with adjustable rates. Comput. Electron. Agric..

[2] Song C., Wang G., Zhao J., Wang J., Yan Y., Wang M., Zhou Z., Lan Y. (2022). Research progress on the particle deposition and distribution characteristics of granular fertilizer application. Trans. Chin. Soc. Agric. Eng..

[3] Zhang C.H., Kovacs J.M. (2012). The application of small unmanned aerial systems for precision agriculture: a review. Precis. Agric..

[4] Jin Z.Y., Guo S.E., Li S.L., Yu F.H., Xu T.Y. (2024). Research on the rice fertiliser decision-making method based on UAV remote sensing data assimilation. Comput. Electron. Agric..

[5] Srivastava K., Pandey P.C., Sharma J.K. (2020). An approach for route Optimization in applications of precision agriculture using UAVs. Drones-Basel.

[6] El Hoummaidi L., Larabi A., Alam K. (2021). Using unmanned aerial systems and deep learning for agriculture mapping in Dubai. Heliyon.

[7] Finger R., Swinton S.M., El Benni N., Walter A., Rausser G.C., Zilberman D. (2019).

[8] Yao W.X., Guo S., Wang J., Chen C.L., Yu F.H., Li X., Xu T.Y., Lan Y.B. (2022). Droplet deposition and pest control efficacy on pine trees from aerial application. Pest Manag. Sci..

[9] Martins R.N., Pinto F.D.D., de Moura A.D., Siqueira W.D., Villar F.M.D. (2020). Nitrogen variable rate fertilization in corn crop prescribed by optical sensor. J. Plant Nutr..

[10] Guerrero A., De Neve S., Mouazen A.M. (2021). Data fusion approach for map-based variable-rate nitrogen fertilization in barley and wheat. Soil Tillage Res..

[11] Shi Y.Y., Chen M., Wang X.C., Odhiambo M.O., Ding W.M. (2018). Numerical simulation of spreading performance and distribution pattern of centrifugal variable-rate fertilizer applicator based on DEM software. Comput. Electron. Agric..

[12] Alameen A.A., Al-Gaadi K.A., Tola E. (2019). Development and performance evaluation of a control system for variable rate granular fertilizer application. Comput. Electron. Agric..

[13] Shi Y.Y., Hu Z.C., Wang X.C., Odhiambo M.O., Sun G.X. (2018). Fertilization strategy and application model using a centrifugal variable-rate fertilizer spreader. Int. J. Agric. Biol. Eng..

[14] Sugirbay A.M., Zhao J., Nukeshev S.O., Chen J. (2020). Determination of pin-roller parameters and evaluation of the uniformity of granular fertilizer application metering devices in precision farming. Comput. Electron. Agric..

[15] Song C.C., Zhou Z.Y., Luo X.W., Jiang R., Lan Y.B., Zhang H.Y. (2018). Review of agricultural materials broadcasting application on unmanned helicopter. Journal of Agricultural Mechanization Research.

[16] Yan X.J., Wang M., Zhu Y.X., Shi X., Liu X.H., Chen Y.X., Xu J., Yang D.B., Yuan H.Z. (2021). Effect of aviation spray adjuvant on improving control of Fusarium head blight and reducing mycotoxin contamination in wheat. Agriculture-Basel.

[17] Chen P.C., Lan Y.B., Huang X.Y., Qi H.X., Wang G.B., Wang J., Wang L.L., Xiao H.X. (2020). Droplet deposition and control of planthoppers of different nozzles in two-stage rice with a quadrotor unmanned aerial vehicle. Agronomy-Basel.

[18] Hussain M., Wang Z., Huang G.M., Mo Y., Kaousar R., Duan L.S., Tan W.M. (2022). Comparison of droplet deposition, 28-homobrassinolide dosage efficacy and working efficiency of the unmanned aerial vehicle and knapsack manual sprayer in the maize field. Agronomy-Basel.

[19] Lou Z.X., Xin F., Han X.Q., Lan Y.B., Duan T.Z., Fu W. (2018). Effect of unmanned aerial vehicle flight height on droplet distribution, drift and control of cotton aphids and spider mites. Agronomy-Basel.

[20] Zhang X.-Q., Song X.-P., Liang Y.-J., Qin Z.-Q., Zhang B.-Q., Wei J.-J., Li Y.-R., Wu J.-M. (2020). Effects of spray parameters of drone on the droplet deposition in sugarcane canopy. Sugar Tech.

[21] Verma A., Singh M., Parmar R.P., Bhullar K.S. (2022). Feasibility study on hexacopter UAV based sprayer for application of environment-friendly biopesticide in guava orchard. J. Environ. Biol..

[22] Wang J., Ma C., Chen P.C., Yao W.X., Yan Y.B., Zeng T.W., Chen S.D., Lan Y.B. (2023). Evaluation of aerial spraying application of multi-rotor unmanned aerial vehicle for Areca catechu protection. Front. Plant Sci..

[23] Martinez-Guanter J., Aguera P., Aguera J., Perez-Ruiz M. (2020). Spray and economics assessment of a UAV-based ultra-low-volume application in olive and citrus orchards. Precis. Agric..

[24] Guo S., Yao W.X., Xu T.Y., Ma H., Sun M.J., Chen C.L., Lan Y.B. (2022). Assessing the application of spot spray in Nanguo pear orchards: effect of nozzle type, spray volume rate and adjuvant. Pest Manag. Sci..

[25] Tang Y., Hou C.J., Luo S.M., Lin J.T., Yang Z., Huang W.F. (2018). Effects of operation height and tree shape on droplet deposition in citrus trees using an unmanned aerial vehicle. Comput. Electron. Agric..

[26] Zakharin F., Ponomarenko S. (2017). 4th IEEE International Conference on Actual Problems of Unmanned Aerial Vehicles Developments (APUAVD).

[27] Zhang S.C., Huang M., Cai C., Sun H., Cheng X.H., Fu J., Xing Q.S., Xue X.Y. (2022). Parameter optimization and impacts on oilseed rape (Brassica napus) seeds aerial seeding based on unmanned agricultural aerial system. Drones-Basel.

[28] Li J.Y., Lan Y.B., Zhou Z.Y., Zeng S., Huang C., Yao W.X., Zhang Y., Zhu Q.Y. (2016). Design and test of operation parameters for rice air broadcasting by unmanned aerial vehicle. Int. J. Agric. Biol. Eng..

[29] Yan X.J., Yuan H.Z., Chen Y.X., Shi X., Liu X.H., Wang Z.Y., Liu Y., Yang D.B. (2022). Broadcasting of tiny granules by drone to mimic liquid spraying for the control of fall armyworm (Spodoptera frugiperda). Pest Manag. Sci..

[30] Zhan Y.L., Chen S.D., Wang G.B., Fu J.W., Lan Y.B. (2021). Biological control technology and application based on agricultural unmanned aerial vehicle (UAV) intelligent delivery of insect natural enemies (Trichogramma) carrier. Pest Manag. Sci..

[31] Latif M.A. (2018). An agricultural perspective on flying sensors state of the art, challenges, and future directions. IEEE Geosci. Remote Sens. Mag..

[32] Song C.C., Zhou Z.Y., Luo X.W., Lan Y.B., He X.G., Ming R., Li K.L., Hassan S.G. (2018). Design and test of centrifugal disc type sowing device for unmanned helicopter. Int. J. Agric. Biol. Eng..

[33] Sreekantha D., Rao K.P.N. (2018). Applications of unmanned ariel vehicles (UAV) in agriculture: a study. Int. J. Res. Appl. Sci. Eng.

[34] Huang X.M., Zhang S., Luo C.M., Li W.C., Liao Y.T. (2020). Design and experimentation of an aerial seeding system for rapeseed based on an air-assisted centralized metering device and a multi-rotor crop protection UAV. Appl. Sci.-Basel.

[35] Xuemei G., Zhaoyan Y., Huichang W., Baoliang P., Shenying W., Mingzhu C. (2022). Design and experiment of green manure seed broadcast sowing device based on unmanned aerial vehicle platform. Nongye Jixie Xuebao/Transactions of the Chinese Society of Agricultural Machinery.

[36] He W., Liu W., Jiang R., Gu Q., Huang J., Zou S., Xu X., Zhou Z. (2022). Control system design and experiments of UAV shot seeding device for rice. Trans. Chin. Soc. Agric. Eng..

[37] Song C.C., Zhou Z.Y., Zang Y., Zhao L.L., Yang W.W., Luo X.W., Jiang R., Ming R., Zang Y., Zi L., Zhu Q.Y. (2021). Variable-rate control system for UAV-based granular fertilizer spreader. Comput. Electron. Agric..

[38] Song C.C., Zang Y., Zhou Z.Y., Luo X.W., Zhao L.L., Ming R., Zi L., Zang Y. (2020). Test and comprehensive evaluation for the performance of UAV-based fertilizer spreaders. IEEE Access.

[39] Su D.X., Yao W.X., Yu F.H., Liu Y.H., Zheng Z.Y., Wang Y.L., Xu T.Y., Chen C.L. (2022). Single-neuron PID UAV variable fertilizer application control system based on a weighted coefficient learning correction. Agriculture-Basel.

[40] Wang R.Y., Zheng Z.A., Lu X.F., Gao L., Jiang D.L., Zhang Z.M. (2021). Design, simulation and test of roller comb type Chrysanthemum (Dendranthema morifolium Ramat) picking machine. Comput. Electron. Agric..

[41] Guo S., Li J.Y., Yao W.X., Hu X.D., Wei X., Long B., Wu H., Li H.F. (2021). Optimization of the factors affecting droplet deposition in rice fields by rotary unmanned aerial vehicles (UAVs). Precis. Agric..

[42] Shi Y.Y., Chen M., Wang X.C., Morice O.O., Li C.G., Ding W.M. (2018). Design and experiment of variable-rate fertilizer spreader with centrifugal distribution cover for rice paddy surface fertilization. Nongye Jixie Xuebao/Transactions of the Chinese Society of Agricultural Machinery.

[43] Fan G.A., Liu Z.J., Qin Y.J., Long B., Li H.F., Li J.Y. (2023). Airflow characteristics of rotorcraft plant protection UAV operating in rice fields. Biosyst. Eng..

[44] Liu Z.J., Fan G.A., Ye S.Y., Zhang Z.X., Wu H., Long B., Li H.F., Cheng H., Wu L.M., Li J.Y. (2023). Flight parameter-wind vortex characteristic control model of a four-multirotor unmanned aerial vehicle operating in pesticide spraying of rice. Agriculture-Basel.

[45] Guo S., Li J.Y., Yao W.X., Zhan Y.L., Li Y.F., Shi Y.Y. (2019). Distribution characteristics on droplet deposition of wind field vortex formed by multi-rotor UAV. PLoS One.

